# Menstrual pattern after abdominal radical trachelectomy

**DOI:** 10.18632/oncotarget.17943

**Published:** 2017-05-16

**Authors:** Xiaoqi Li, Jin Li, Xingzhu Ju, Zhaoxia Jiang, Xiaojun Chen, Xiaohua Wu

**Affiliations:** ^1^ Department of Gynecologic Oncology, Fudan University Shanghai Cancer Center, Shanghai, PR China; ^2^ Department of Radiology, Fudan University Shanghai Cancer Center, Shanghai, PR China

**Keywords:** abdominal radical trachelectomy (ART), cervical cancer, menstrual pattern, cervical stenosis

## Abstract

To assess changes of menstrual patterns, their causes, preventions and treatment methods after abdominal radical trachelectomy (ART), we recorded patients’ menstrual patterns after surgery and compared them with the conditions before surgery. Patients were divided into 3 groups based on their menstrual status post-trachelectomy:Group 1: menstrual patterns unchanged; Group 2: menstrual patterns changed without amenorrhea; and Group 3: amenorrhea. One hundred and twenty-nine patients were included: 39 (30.2%) women belonged to Group 1, 74 (57.4%) to Group 2 and 16 (12.4%) to Group 3. For patients in Group 2, the most common presenting symptom was decrease of menstrual volume (59, 79.7%), followed by a prolonged (33, 44.6%) menstrual bleeding. All of the changes in Group 2 and 12 cases in Group 3 were due to narrow of the remaining cervical os. Additionally, 9 and 12 patients, respectively, in Group 2 and 3, developed cervical stenosis. To maintain regular menstruation and prevent isthmic stenosis, 99 patients had tailed intrauterine devices (IUDs) placed in the uterine cavity. Incidence of cervical stenosis all happened in patients without IUDs placed in the uterine cavity. Menstrual condition improved in all patients except one after dilation of the new-cervix. Our results demonstrated that the majority of patients suffered from changes in menstrual patterns after ART. Narrowing of the remaining cervical canal was the main reason and could be treated by cervical dilation. The use of a tailed IUD was effective in the prevention of this complication.

## INTRODUCTION

Radical trachelectomy (RT) in combination with pelvic lymph node dissection is now regarded as an alternative to radical hysterectomy and is increasingly being offered to selected women with early stage cervical carcinoma who wish to preserve fertility. Several studies have been published and have proved the oncologic safety as well as the effectiveness in the maintenance of fertility potential of this procedure [1–4]. However, changes in menstrual patterns, which are unique postoperative manifestations that might impact patients’ quality of life and obstetrical outcomes following radical trachelectomy, have not been thoroughly investigated.

Limited data exist describing chemotherapy and cervical stenosis as the cause of amenorrhea after RT [3, 5, 6]. Additionally, few studies have mentioned decreased menstrual flow and/or dysmenorrhea after RT compared with status preoperatively [5, 7]. However, no study has specifically investigated the incidences, risk factors, prevention and treatment methods of such a complication. Because amenorrhea is associated with infertility, and changes of menstruation can increases the risk of anxiety and discomfort, better understanding of the menstrual changes after RT would facilitate patients counseling about fertility options and help improve patients’ quality of life.

Concerning the background provided above, we conducted the present study to record patients’ menstrual patterns after abdominal radical trachelectomy (ART) and compared them with the conditions before surgery, in order to (1) describe menstrual status in women who underwent ART, (2) determine risk factors that lead to the menstruation changes, and (3) explore the prevention and treatment methods.

## RESULTS

Between April 2004 and May 2015, 160 patients with early stage cervical cancer underwent ART by the same group of surgeons at our institution. Fourteen patients were withdrawn: 9 patients had pathological types of botryoid sarcoma or adenosarcoma; 3 patients experienced recurrence and 2 received radiotherapy due to positive lymph nodes at final pathology. One hundred and twenty-nine patients completed our survey and were included in the present study. Demographic and pathologic outcomes did not differ significantly between the participants and non-participants. With a median follow-up of 33 months (range 6–123), 39 (30.2%) women belonged to Group 1, 74 (57.4%) to Group 2 and 16 (12.4%) belonged to Group 3. Patients’ characteristics and pathologic outcomes were summarized in Table 1.

**Table 1:** Demographic and pathologic outcomes of the participants

	Group 1 N=39 (30.2%)	Group 2 N=74 (57.4%)	Group 3 N=16 (12.4%)	
**Age at diagnosis (years), Mean (range)**	32 (20-42)	32 (21-43)	33 (26-42)	NS
**Age at last follow-up, (years), Mean (range)**	35 (22-45)	35 (26-43)	37 (31-45)	NS
**BMI (kg/m2), Mean (range)**	21.3 (16.6-28.0)	21.4 (16.6-33.9)	20.7 (16.4-26.0)	NS
**Marital status, N(%)**				NS
**Married**	30 (76.9%)	62 (83.8%)	13 (81.3%)	
**Unmarried**	9 (23.1%)	12 (16.2%)	3 (18.7%)	
**Parity before the surgery, N (%)**				NS
**0**	24 (61.5%)	39 (52.7%)	10 (62.5%)	
**1**	15 (38.5%)	26 (35.1%)	6 (37.5%)	
**2**	0	9 (12.2%)	0	
**FIGO stage, N (%)**				NS
**IA1**	5 (12.8%)	8 (10.8%)	4 (25.0%)	
**IA2**	4 (10.3%)	7 (9.5%)	2 (12.5%)	
**IB1**	30 (76.9%)	59 (79.7%)	10 (62.5%)	
**Tumor size (cm), Mean (range)**	1.8 (0.1-4.0)	1.5 (0.1-4.0)	1.2 (0.3-3.0)	NS
**Histology, N (%)**				NS
**Squamous**	34 (87.2%)	60 (81.1%)	14 (87.5%)	
**Adenocarcinoma**	4 (10.3%)	11 (14.9%)	2 (12.5%)	
**Adenosquamous**	1 (2.5%)	3 (4.0%)	0	
**Prior LEEP or cone, N (%)**	15 (38.5%)	31 (41.9%)	11 (68.8%)	NS
**Adjutant Chemotherapy, N (%)**	8 (20.5%)	17 (23.0%)	2 (12.5%)	NS
**Cervical stenosis, N (%)**	0	9 (12.2%)	12 (75.0%)	<0.001
**Follow-up time (months), Median (range)**	33 (7-123)	30(6-104)	47 (16-106)	0.047

BMI, Body Mass Index; FIGO, International Federation of Gynecology and Obstetrics; LEEP, Loop Electrosurgical Excision Procedure.

### Menstruation changes and the causes

For patients in Group 1, no one complained of obvious changes of lifestyle habits and mental stresses after surgery. All were detected with normal basal body temperatures, normal basal hormone levels and undamaged endometrium during the follow-up time. The cervix os were all clearly observed by gynecological examination.

For patients in Group 2, the most common presenting symptom was a decrease in the volume of menstrual fluid (59, 79.7%), with 31(41.9%) patients reporting a slight decrease in menstrual volume, 23 (31.1%) reporting an moderate decrease and 5 (6.7%) reporting an obvious decrease. Secondly, patients reported a change of bleeding length (46, 62.2%), including 33 (44.6%) women who presented with a prolonged menstrual bleeding and 13 (17.6%) who presented with shortened menstrual bleeding, followed by newly developed dysmenorrhea (19, 25.7%). Forty-three (58.1%) patients had more than one change. Menstrual cycle lengths only changed in 3 of the patients.

The majority of patients (58, 78.4%) complained about menstruation changes during the first one or two cycles following surgery, and remained relatively constant during the follow-up period. Nine (12.2%) patients experienced gradual menstruation changes over several months or years. Five (6.7%) patients experienced changes after the removal of the IUD, and 2 (2.7%) after chemotherapy.

None of the patients complained of an obvious changes of lifestyle habits and mental stresses after surgery. None had abnormal basal body temperatures, abnormal basal hormone levels and damaged endometrium. All were detected with narrow or even pinpoint-sized cervical os with or without vaginal mucosa encroachment by gynecological examination. Nine patients had cervical stenosis and 7 underwent cervical dilation: 4 women experienced an increase in menstrual volume, 2 had a shortened bleeding duration and 1 had dysmenorrhea disappeared after dilation of the new cervix.

For 16 women in Group 3, one (6.3%) became postmenopausal at the age of 42 years as a result of ovarian dysfunction after 6 cycles of chemotherapy with paclitaxel and cisplatin. Three patients had intrauterine adhesion: one (6.3%) experienced severe fibrosis of the basal layer of endometrium and did not resume menstruation even after cervical dilation, adhesiolysis and hormone therapy; two (12.4%) had endocervical and intrauterine adhesion and recovered regular menstruation naturally 1 and 3 years after the operation, respectively. The remaining 12 (75.0%) patients’ changes were due to cervical stenosis.

Among those 12 patients who had cervical stenosis, 4 did not resume menstruation after ART: 2 did not insert an IUD during the surgery, and 2 had the IUD removed 1 week after surgery. Eight maintained regular periods after surgery with an IUD placed and then experienced ceased menstruation one month to 4 years after the removal of the IUD (7 patients) or after the tail of the IUD dropped (1 patient). Gynecological examination showed a total block of the cervical os with or without hematometras detected by transvaginal ultrasonography. Five patients complained of a dysmenorrhea. Eleven women underwent cervical dilation and 10 resumed monthly bleeding except 1 patient whose first dilation failed and further treatment was refused.

### Preventive measures

To maintain regular menstruation and prevent cervical stenosis after surgery, we chose to place a Foley catheter (Cath, Shandong, China) during the early stages of our study. The catheter was inserted in the uterine cavity with the balloon portion filled with 5 mL saline and the end fixed in the endocervical canal. However, this device was less comfortable and could easily fall out upon ambulation. In total, 10 patients had the catheter placed during the operation and all complained of discomfort. Nine of the catheters dropped down automatically and 1 was removed due to bleeding. Median duration time was 10 (1-40) days. Two patients (20.0%) developed cervical stenosis 1 and 2 years, respectively, after the catheters fell out.

For the sake of comfort and long duration time, we now routinely utilize a tailed IUD during the operation. The IUD was placed in the uterine cavity with a tail preserved 2-3 cm standing outside of the new-cervical os. Thus far, 99 patients had the IUD placed during the operation and 14 patients (14.1%) developed cervical stenosis. Details of the placement of the IUD and occurrence time of cervical stenosis were shown in Table 2. As was shown in the table, patients who had no tailed IUD placed during the surgery experienced more chance of developing cervical stenosis compared to these with tailed IUDs placed (23.2% vs. 14.1%). Additionally, all of the patients with tailed IUDs placed during the surgery suffered from cervical stenosis after the tailed IUD removed or the tail dropped. In other words, all of the cervical stenosis happened when patients did not have a tailed IUD placed in the uterine cavity.

**Table 2:** Placement of the tailed intrauterine device (IUD) and the occurrence time of cervical stenosis

Placement of the tailed IUD during surgery	No, total number of the patients (number of the patients with cervical stenosis)	Yes, total number of the patients (number of the patients with cervical stenosis)	
**Occurrence time of cervical stenosis**		A tailed IUD not placed in the uterine cavity^1^	A tailed IUD placed in the uterine cavity
**Group 1**	10 (0)	23(0)	6 (0)
**Group 2**	18 (5)	38 (4)	18 (0)
**Group 3**	2 (2)	14 (10)	0 (0)

1. This includes the conditions when the tailed IUD removed or only the tail of the IUD dropped.

The IUD was removed 3-6 months after surgery during the early stages of our study. Because several patients complained about stopped menstruation after removal of the IUD, we now prefer to keep the device in place until patients either attempt to conceive or develop IUD related complications. So far, 75 patients have had the device removed. Fifty-seven (76.0%) were removed in order to conceive. Nine (12.0%) dropped automatically. Six (8.0%) patients suffered from chronic discharge and 3 (4.0%) experienced bleeding related to the IUD. All symptoms resolved spontaneously after removal of the IUD. The median duration time was 8 (5-102) months.

**Figure 1:**
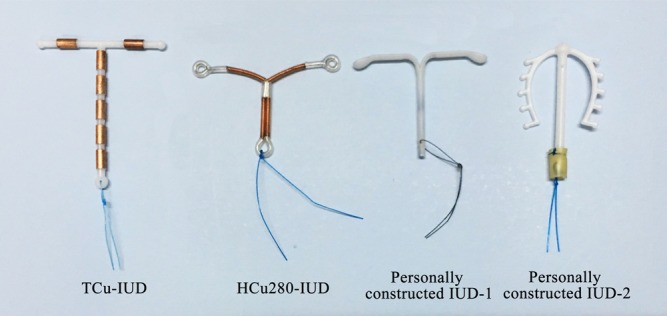
Types of the tailed intrauterine devices (IUDs) used at our institution Personally constructed IUD-1: a tailed plastic IUD with the copper components and part of the vertical arm moved and a wire tied at the end of the IUD. Personally constructed IUD-2: a tailed plastic IUD with the end of the vertical stem connected to a plastic tube.

The types of the tailed IUDs we used were shown in Figure 1. At first, we chose a tailed T-shaped copper containing intrauterine device (TCu-IUD) (Tianyi, Wuxi, China). However, the stem of that device was too long to stand outside of the external os which increased discomfort during intercourse. After searching for a much shorter device, we discovered the tailed HCu280-IUD (Xinxin, Shenyang, China). However, the copper components covered on the surface of the IUD brought inconvenience to follow-up examination. Patients either had to remove the IUD prior to undergoing pelvic magnetic resonance imaging (MRI) or had pelvic ultrasound or computed tomography (CT) performed instead, which are not as clear as MRI. Based on the reasons above, we personally constructed a tailed plastic IUD with the copper components and part of the vertical arm moved and a wire tied at the end of the IUD (Patent NO.: CN201420660489.0). Recently, we further constructed the IUD with the end of the vertical stem connected to a plastic tube (Patent NO.: CN201620441214.7). We hope this device will better expand the cervical canal and prevent the incidence of cervical stenosis.

Thus far, 18 patients experienced cervical stenosis and obviously changed menstruation or amenorrhea underwent cervical dilation at our institution. Among the 7 patients who suffered from cervical stenosis while still maintaining regular menstruation, 4 women complained of an increase in menstrual volume, 2 had a shortened bleeding duration and 1 had dysmenorrhea disappeared after dilation of the new cervix. Among 11 patients who experienced absence of menstrual periods after ART, all resumed monthly bleeding after cannulation except 1 patient whose first dilation failed and then refused further treatment.

**Figure 2:**
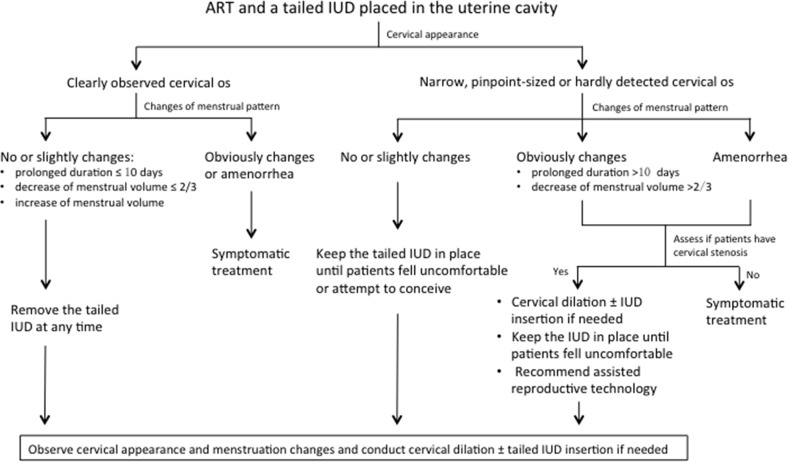
Treatment guideline to maintain regular menstruation with the use of a tailed IUD

However, dilation had to be conducted several times until re-insertion of an IUD to obtain optimal results. In our study, 1 woman underwent 5 dilations, 2 women underwent 4 dilations, 3 had three dilations, 3 had two dilations and 9 had one dilation. Five of these were managed with in-office dilation and 13 required general anesthesia in the operating room with or without ultrasound guidance. One patient's procedure was conducted using an electric knife to excise the stenotic segment after failure of transvaginal cannulation. Three women underwent transfundal cervical dilation. Diagnosis and treatment guideline on post-ART menstruation changes and the use of IUD at our institution were shown in Figure 2.

## DISCUSSION

The management of fertility-sparing surgery for young patients with early stage cervical cancer must find an appropriate balance among oncological safety, obstetric outcomes, and complications after surgery. Changes of menstruation are specific complications after ART that might affect not only quality of lives but also patients’ reproductive function. Thus, this requires special attention.

The majority of patients (88, 68.2%) experienced narrow of the remaining cervical canal due to formation of the cervical scar, cervical canal adhesions and the encroachment of vaginal mucosa over the neo-cervix. The constriction of the remaining cervical canal could impede menstrual outflow thereby prolonging the duration of bleeding length. Additionally, it could limit the growth of normal endometrium [9, 10] and increase retrograde menstruation [11], thus resulting in the reduction of menstrual volume. The increased pressure of the uterine cavity itself as well as the resulting risk of endometriosis all contributed to the development of pelvic pain after surgery [11]. This process took roughly one or two months based on the fact that most patients complained about changes of menstruation pattern during the first one or two cycles following surgery, and these changes remained constant thereafter. In the majority of patients (53.5%), the remaining cervical canal became narrowing and they did not require any treatment. However, 16.2% of the patients experienced cervical stenosis and suffered from great changes of menstrual pattern or even amenorrhea thereby need cervical dilation as soon as possible. Our study was in concordance with previous reported finding by Carter el al.[12] that 73.3% of the patients were noted to have some vaginal scarring, encroachment, and/or stenosis after radical trachelectomy. Because stenosis of the new cervix could also increase dyspareunia [12] and is a potential cause of infertility requiring consideration [3, 13], better prevention of cervical stenosis could help decrease patients’ discomfort and increase chances of getting pregnant.

The use of a tailed IUD could effectively reduce the occurrence of cervical stenosis. The thread placed in the cervical canal could prevent cervical scar formation, cervical canal adhesions and the encroachment of vaginal mucosa over the neo-cervix. Incidences of stenosis all happened in patients without tailed IUDs placed during the surgery or after the device or the tail removed. Therefore, we highly recommend the routinely utilization of a tailed IUD, especially the custom-made tailed copper free containing IUD placed during the procedure. Although cervical dilation is an effective method to treat isthmic stenosis, it is painful and tedious. Some patients require several dilations before the issue is resolved. In order to maintain regular menstruation, prevent the incidence of cervical stenosis, and decrease the chance of cannulation, we routinely placed a tailed IUD in the uterine cavity during the ART procedure. Instruction on when to remove the device is shown in Figure 2.

The definition of cervical stenosis varies form article to article. Several authors defined it as sonographic evidence of haematometra and/or amenorrhea requiring cervical dilation [14]. This definition ignored cases in which menstrual flow continued to occur but os identification was difficult or impossible thus need treatment. Others defined cervical stenosis as a standard endocervical brush could not be easily introduced into the os without mechanical dilation [12, 15, 16]. This broader definition included patients with slight changes of menstruation that do not need any treatment. In order to easily distinguish patients with cervical issue who need cervical cannulation, we preferred to use menstrual status to diagnose isthmic stenosis. Because, based on our experience, cervical factors were the main cause of menstruation changes after ART, and patients with prolonged duration of menses by more than 10 days, with obvious decreased menstrual volume by greater than 2/3 or even amenorrhea need the treatment most.

Chemotherapeutic agents have an effect on ovarian function and may potentially impair menstrual cycle and reproductive function. Taxol and platinum (TP) are viewed to be only moderately gonadotoxic agents that have less devastating effects on oocytes and menstruation [17, 18]. In our study, patients who received chemotherapy did not differ significantly among the three groups. However, age is an important determinant of ovarian failure after chemotherapy, incidence of amenorrhea increases significantly after age 42 [19, 20]. In our study, even though the majority of patients maintained regular menstruation after surgery, one woman at the age of 42 experienced ovarian dysfunction after administration of 6 cycles of TP regimen. Based on the reasons above, eligible patients over 42 years of age should be fully assessed concerning the possibility of undergoing adjuvant therapy and informed about the potential damage of ovarian reserve as well as reproductive function after chemotherapy prior to the start of the surgery.

To the best of our knowledge, this is the first study describing changes of menstrual pattern, the causes, prevent and treatment methods following ART. Our study provides a guideline in the maintenance of regular menstruation as well as prevention and treatment of cervical stenosis after ART based on our 10 years of experience. However, there still exist some limitations due to its retrospective nature. Further prospective studies are needed to reinforce these results.

## CONCLUSIONS

The majority of patients experienced decreased menstrual volume, prolonged or shortened bleeding length, and developed new dysmenorrhea due to narrowing of the remaining cervical canal following ART. 16.3% of the patients would develop cervical stenosis and suffer from great changes of menstrual patterns or even amenorrhea. The use of a tailed IUD could help maintain a regular menstruation and is effective in the reduction of stenosis, thereby is highly recommended during the procedure. Dilation is a very simple and helpful procedure for the management of cervical stenosis and can also improve the condition of menstrual status. Age is another risk factor of amenorrhea after chemotherapy, eligible patients over 42 years old should be fully informed about the possibility of ovarian failure after chemotherapy.

## MATERIALS AND METHODS

From July 2014 to December 2015, we recorded menstrual patterns of patients who underwent ART and compared them with the conditions before surgery. Eligible patients were those who: (1) underwent ART by the same group of surgeons; (2) had a pathological type of squamous carcinoma, adenocarcinoma or adenosquamous carcinoma; (3) did not receive adjuvant radiotherapy after surgery; (4) had been disease-free without recurrence during the follow-up; (5) maintained regular menstruation and did not have ovarian, hypothalamus–pituitary, thyroid or other lesions that could cause menstrual problem before ART; (6) had a follow-up time≥ 6 months.

The study had been reviewed and approved by the ethics committee of Fudan University Shanghai Cancer Center. All patients gave written informed consent to participate in the study.

Eligible patients were asked the condition of their menstruations at the first time of the follow-up time. If patients experienced stopped menstruation after surgery, they were asked to record details of the ceased menstruation, including occurrence time, causes, treatments and outcomes. If patients never experienced cessation of menstruation, they were required to record details of their menstruation for six months, including menstrual duration, cycle length, dysmenorrhea and menstrual blood volume. If patients received treatments, such as cervical dilation or hormone therapy during the follow-up time, a detailed record of the menstruation for at least three months after treatment was also required. All of the above information were confirmed and supplemented by the prospectively recorded database. Patients’ menstrual patterns before surgery were also acquired from this database. After the follow-up, patients were required to complete a questionnaire with the help of the doctors. See Supporting Information 1.

Menstrual duration and cycle length were the average value of each record. Dysmenorrhea was assessed by the verbal multidimensional scoring system, which grades menstrual pain as none, mild, moderate or severe [8]. Change in menstrual blood volume was estimated according to the number and saturation degree of the used menstrual pads. Less than or equal to a 1/3 reduction or increase in menstrual volume was regarded as slight decreased/increased; one third to 2/3 alterations of blood loss were considered as moderate change; and greater than 2/3 changes were considered as obvious change. If changes of blood volume and dysmenorrhea condition were different at each menstruation, we would choose the one with the highest frequency as the final result.

### Grouping

Patients in our study were divided into 3 groups based on their menstrual status post-trachelectomy: Group 1 “menstrual pattern unchanged”: patients’ menstrual pattern in terms of duration, cycle length, severity of menstrual pain, and volume did not change after surgery; Group 2 “menstrual pattern changed without amenorrhea”: patients’ menstrual pattern changed compared to the condition before surgery but did not ceased; and Group 3 “amenorrhea”: patients experienced absence of menstrual periods after ART. Changes of menstruation were decided according to the condition before treatment. Changes of cycle length within one week were regarded as no changes.

### Diagnosis of menstruation changes

For patients either experiencing changes of menstruation or a halt in menstruation following ART, a detailed medical history, including stress, dieting, and strenuous exercise was carefully obtained. Gynecologic examination included cervical appearance and/or pelvic ultrasound of the endometrial state and observation of hematometra were conducted to exclude incidence of cervical stenosis and/or endometrial injury. Measurement of basal body temperature; hormone assessments of E2 (estrogen), P (progestin), FSH (follicle-stimulating hormone), LH (luteinizing hormone), AMH (anti miillerian hormoneor); or diagnostic hormonotherapy were necessary to rule out ovarian, pituitary, and hypothalamic dysfunctions. Hysteroscopy was performed if needed.

The definition of cervical stenosis is a condition that leads to prolonged duration of menses by more than 10 days, obviously decreased menstrual volume by greater than 2/3 or even amenorrhea due to cervical issues only after exclusion of endometrial, ovarian, hypothalamus-pituitary and other factors takes place.

### Cervical dilation

Cervical dilation was usually conducted if patients presented with isthmic stenosis. Cannulation took place on an outpatient basis with or without local anesthesia, and was generally started during menstruation with a dilator ranging from a 1 mm mini Hegar dilator up to a 7 mm dilator. If the in-office dilation failure occurred, cannulation was scheduled in the operating room under intravenous anesthetics and/or ultrasound guidance to avoid creation of a false passage. Once a 5 mm Hegar dilator was easily advanced into the neo-cervix, a tailed intrauterine device (IUD) or a Foley catheter could be inserted and left in place to prevent re-stenosis. If the transvaginal cannulation failed, use of an electronic knife to excise the stenotic scar tissue and clear the cervical canal would be conducted for the outflow of the menstrual blood. In several patients, for whom the residual cervix was too small to perform a transvaginal procedure, the laparotomy was elected, where a 1.5 cm-long vertical incision was made at the lower part of the front uterine wall, and the dilator was placed in the uterine cavity, expanding the cervix from top to bottom.

### Statistical analysis

Statistical analysis was performed using the SPSS 21.0 software (Chicago, IL, USA). Categorical variables were described with proportions and continuous variables with the mean/median and range. The associations between the categorical data were assessed using χ2 test. Fisher's exact test was used when it was necessary. Continuous variables were analyzed by ANOVA test or t test when appropriate. P values less than 0.05 were considered statistically significant.

## SUPPLEMENTARY SUPPORTING INFORMATION




