# Supplementary material for: Menstrual pattern after abdominal radical trachelectomy
Source: Oncotarget. 2017 May 16;8(32):53146–53. doi: 10.18632/oncotarget.17943 (PMC5581099; doi:10.18632/oncotarget.17943)
Supplement: Supplementary file 1 [file oncotarget-08-53146-s001.pdf]

Menstrual pattern after abdominal radical trachelectomy

SUPPLEMENTARY SUPPORTING INFORMATION

Supporting Information 1: Questionnaire of the menstrual pattern in patients undergoing ART.

See Supplementary File 1
