# Supplementary material for: Menstrual pattern after abdominal radical trachelectomy
Source: Oncotarget. 2017 May 16;8(32):53146–53. doi: 10.18632/oncotarget.17943 (PMC5581099; doi:10.18632/oncotarget.17943)
Supplement: Supplementary file 2 [file oncotarget-08-53146-s002.docx]

Date:

Check (√) and complete where applicable.

**Menstrual pattern of patients undergoing ART**

**1. Basic information**

Name: Age: ID : Telephone:

Marriage：□single □married □divorced □remarried □widowed

Number of children before surgery: □0 □1 □2 □3

Height: m Weight: kg BMI: kg/m^2^

Date of operation:

**2. Preoperative menstrual pattern**

Duration: days

Cycle length: days

Dysmenorrhea: □no □yes

Degree of dysmenorrhea: □none □mild □moderate □severe

The verbal multidimensional scoring system (VMS), (evaluation standard of menstrual pain):

- None: menstruation is not painful and daily activity is unaffected
- Mild: menstruation is painful but seldom inhibits the woman’s normal activity. Analgesics are seldom required.
- Moderate: daily activity affected. Analgesics required and provide relief so that absence from work or school is unusual.
- Severe: activity clearly inhibited. Poor effect of analgesics. Vegetative symptoms, e.g., headache, tiredness, nausea, vomiting and diarrhea.

**3. Adjuvant therapy**

□none □chemotherapy

Date of last adjuvant therapy:

Cycles of chemotherapy: cycles, regimen:

Changes of menstrual pattern after chemotherapy: □none □changed, (please detail the changes)

**4. Postoperative menstrual pattern**

Duration: days (the average duration days of each menstrual cycle)

Cycle length: days (the average length of cycles of each menstrual cycle)

Dysmenorrhea: □no □yes

Degree of dysmenorrhea: □none □mild □moderate □severe

Changing trend of dysmenorrhea: □none □worse □better

Change in menstrual blood volume:

□none

□slightly decreased □slightly increased

□moderate decreased □moderate increased

□obviously decreased □obviously increased

Note:

Slightly: ≤1/3 decrease/increase

Moderate: 1/3-2/3 decrease/increase

Obviously: >2/3 decrease/increase

**5. Anti-stenosis tools**

Was an anti-stenosis tool placed in the uterine cavity during surgery?

□no(skip to question 6) □yes

Which tool it is? □tailed intrauterine device (IUD) □catheter

Is it removed? □no □yes

How long has it been placed?

Reasons of remove:

□attempt to conceive □drop down automatically

□lead to bleeding □lead to infection □others,

Changes of menstrual pattern after removal of the tool: □none □changed, (please detail the changes)

**6. Cervical dilation**

Have you undergoing cervical dilation? □no(skip this section) □yes

Number of dilations: times

Please detail the exact time, location (in the office or in the operating room), maximum diameter of Hegar dilator used (1-7mm), placement of the anti-stenosis tool after dilation or not, and changes of menstrual pattern after dilation.

**This is the end of the questionnaire.**

**Thank you very much for your patience and sincere cooperation.**
